# Blocking Spatial Navigation Across Environments That Have a Different Shape

**DOI:** 10.1037/xan0000084

**Published:** 2015-11-16

**Authors:** Matthew G. Buckley, Alastair D. Smith, Mark Haselgrove

**Affiliations:** 1 School of Psychology, University of Nottingham

**Keywords:** spatial learning, geometric module, blocking, cue competition

## Abstract

According to the geometric module hypothesis, organisms encode a global representation of the space in which they navigate, and this representation is not prone to interference from other cues. A number of studies, however, have shown that both human and non-human animals can navigate on the basis of local geometric cues provided by the shape of an environment. According to the model of spatial learning proposed by Miller and Shettleworth (2007, 2008), geometric cues compete for associative strength in the same manner as non-geometric cues do. The experiments reported here were designed to test if humans learn about local geometric cues in a manner consistent with the Miller-Shettleworth model. Experiment 1 replicated previous findings that humans transfer navigational behavior, based on local geometric cues, from a rectangle-shaped environment to a kite-shaped environment, and vice versa. In Experiments 2 and 3, it was observed that learning about non-geometric cues blocked, and were blocked by, learning about local geometric cues. The reciprocal blocking observed is consistent with associative theories of spatial learning; however, it is difficult to explain the observed effects with theories of global-shape encoding in their current form.

There is now considerable evidence to show that navigation based upon landmarks is consistent with an associative explanation of spatial learning (e.g., Chamizo, Aznar-Casanova, & Artigas, 2003; Chamizo, Manteiga, Rodrigo, & Mackintosh, 2006; Gould-Beierle & Kamil, 1999; Leising, Garlick, & Blaisdell, 2011; Roberts & Pearce, 1999; Sanchez-Moreno, Rodrigo, Chamizo, & Mackintosh, 1999; Stahlman & Blaisdell, 2009). There remains, however, much debate about how learning about the shape properties of an environment progresses. The origins of this debate can be traced to experiments by Cheng (1986), in which rats were trained to find food buried in a rectangular arena that contained a unique feature cue in each corner. Food was hidden, for example, in a corner formed by the joining of a short wall to the left of a long wall (Corner W, Figure 1). Navigating on the basis of shape-information alone would lead rats to the corner containing the buried food, or the diametrically opposite corner of the rectangle (Corner Z, Figure 1). Consequently, the shape of the arena provided an ambiguous cue for learning. In contrast, the features present at each corner provided unambiguous cues for the location of the food. When the features were removed from the arena, the rats continued to search in the correct, or geometrically equivalent, corners more often than would be expected by chance. Consequently, the rats had clearly learned about the location of the food with respect to the ambiguous shape-information, despite the presence of a better predictor of the food’s location. These, and similar results (e.g., Margules & Gallistel, 1988), have led to the suggestion that organisms encode a *global* representation of the shape of their environments in a dedicated geometric module that is impervious to the influence of non-geometric cues (Cheng, 1986; Gallistel, 1990; Wang & Spelke, 2002).[Fig-anchor fig1]

The notion that animals encode a global representation of the shape of their environments has not gone unchallenged. As noted by Pearce, Good, Jones, and McGregor (2004), in order to find the buried food, rats in Cheng’s experiment need not have learned anything about the global shape of the environment. Instead, rats could have learned only about the individual corner in which the food was buried. That is, following the previous example, rats could have simply learned to approach a corner where the left-hand wall was shorter than the right-hand wall, which would lead to rats searching in the corner of the arena that contained the buried food (Corner W, Figure 1), or the geometrically equivalent corner (Corner Z, Figure 1). This explanation for how organisms use the geometry of their environment to find a hidden goal can be referred to as a *local* solution. Pearce et al. (2004) provided evidence consistent with the notion that rats navigate on the basis of local shape-information by training them to find a submerged platform in a particular corner of a kite-shaped arena that contained two right-angled corners, before placing them in a rectangle-shaped arena. If, for example, rats were trained to navigate to the right-angled corner of a kite where the left-hand wall was shorter than the right-hand wall (Corner A, Figure 1), they preferentially searched in the corners of the rectangle that shared the same local geometric cues (Corners W and Z, Figure 1). This behavior is difficult to reconcile with the notion that rats navigate only on the basis of a global representation of the shape of their environment, as the global shape of the environment changed between training and testing (see also Lew et al., 2014; McGregor, Jones, Good, & Pearce, 2006; Poulter, Kosaki, Easton, & McGregor, 2013; Tommasi & Polli, 2004).

One manner in which learning about local shape-information might proceed is according to the model of spatial navigation provided by Miller and Shettleworth (2007, 2008, 2013). According to this model, both geometric and non-geometric cues are encoded in an elemental fashion. These elements then compete for an association with a navigational goal according to Equation 1.

$$\Delta {\text{V}}_{\text{E}}=\;\alpha \;\left(\lambda -{\text{V}}_{\text{L}}\right)P_{\text{L}}$$ 1

Equation 1 is a modification of the learning rule proposed by Rescorla and Wagner (1972). V_E_ denotes the strength of the association between an element (e.g., a particular corner of an environment) and the navigational goal, α denotes the associability of that element, λ denotes the asymptote of learning supported by the navigational goal, and V_L_ denotes the sum of the associative strengths of all elements at a particular location. The addition of *P*_L_ to the Rescorla-Wagner model denotes the probability of choosing a particular location within an environment, which itself is defined as:

$$P_{\text{L}}={\text{V}}_{\text{L}}/\Sigma {\text{V}}_{\text{L}}$$ 2

As before, V_L_ is the associative strength of all elements at a particular location, and ΣV_L_ is the sum of the associative strengths of all locations. By using the Rescorla-Wagner learning algorithm as a starting point, and incorporating *P*_L_ into the learning equation, the model proposed by Miller and Shettleworth (2007, 2008, 2013) can successfully simulate the results of a number of experiments where non-geometric cues have successfully blocked learning about geometric information (e.g., Horne & Pearce, 2009; Pearce, Graham, Good, Jones, & McGregor, 2006; Wilson & Alexander, 2008). Moreover, the Miller-Shettleworth model also provides a basis for understanding experiments in which non-geometric cues have failed to block learning about geometry information (e.g., Hayward, Good, & Pearce, 2004; Hayward, McGregor, Good, & Pearce, 2003; Pearce, Ward-Robinson, Good, Fussell, & Aydin, 2001; Redhead & Hamilton, 2009; Wall, Botly, Black, & Shettleworth, 2004).

Consider an experiment in which an animal is initially trained to locate a hidden goal on the basis of only a non-geometric cue (e.g., a landmark), after which it is placed into a novel arena in which the hidden goal can be located on the basis the geometry of the environment as well as the original non-geometric cue. The model proposed by Miller and Shettleworth (2007) explains blocking in a similar manner to the Rescorla-Wagner model on which it is based. The associative strength of the non-geometric cue that signals the goal location in Stage 1 will approach asymptote and, thus, prevent the geometric cues gaining any associative strength when they are introduced in Stage 2. The blocking effect, however, can be undermined by a process Miller and Shettleworth (2007) termed feature enhancement. During Stage 1 training, the probability choice rule described in Equation 2 ensures that the animal consistently approaches the non-geometric cue that signals the goal location long before the associative strength of the non-geometric cue reaches asymptote. Consequently, if only minimal Stage 1 training is administered, at the onset of Stage 2 the animal will consistently approach the non-geometric cue, permitting the associative strength of the correct geometry to increase quicker than would normally be expected. Relative to an appropriate control group, therefore, learning about environmental geometry would appear unimpaired in the blocking group.

Despite providing a compelling explanation for spatial learning phenomena, the model proposed by Miller and Shettleworth (2007, 2008, 2013) does not explicitly state how organisms encode geometric information. On the basis of the evidence reviewed above, it is not unreasonable to expect that it is *local* geometric information that competes with other navigational information, such as feature cues, in order for effects like blocking to be observed. It is, however, difficult to find evidence that supports this notion, despite the aforementioned observations that appear consistent with this prediction. In Stage 1 of Experiment 4 conducted by Pearce et al. (2006), rats were placed in a square arena comprising two adjacent back walls and two adjacent white walls, and trained to find a hidden platform in the corner where the two black walls joined. Subsequently, rats were placed into a rectangular arena that also comprised two adjacent black walls and two adjacent white walls, and were again trained to swim to a submerged platform. For rats in an experimental group, the platform was located in the all-black corner which had a short wall to the left of a long wall. For rats in a control group, the platform was located in the all-white corner that had a short wall to the left of the long wall. During test trials conducted without the platform, rats were placed into a rectangular arena, the walls of which were all the same color. While the rats in the control group displayed a significant preference for the corners of the rectangle where a short wall was to the left of a long wall (Corners W and Z, Figure 1), rats in the experimental group displayed no preference. These results, then, demonstrate a blocking effect. In the experimental group, learning about the non-geometric wall color information in Stage 1 prevented learning about the shape-information in Stage 2, a result consistent with the model of spatial navigation provided by Miller and Shettleworth. The test trials, though, were conducted in an environment that was the same shape as the environment from Stage 2 training. Consequently, it is not at all clear whether learning about the wall colors in Stage 1 of the experiment blocked learning about the *local* geometric features of the environment in Stage 2 or, instead, learning about the *global* shape of the Stage 2 environment.

The experiments reported here were designed to assess if local geometric cues compete with non-geometric cues in manner consistent with the Miller-Shettleworth model. The purpose of Experiment 1 was to establish parameters with which, in our laboratory, learning that is based about the shape of one environment (e.g., a rectangle) transfers to a different-shaped environment (e.g., a kite). In Experiments 2 and 3, we applied the procedures from Experiment 1 to assess if the geometric information that is transferred between environments that have a different shape can be blocked by (Experiment 2), or block (Experiment 3), learning about non-geometric wall color information.

## Experiment 1

In Experiment 1, participants in group kite-rectangle were trained to find a hidden goal in one of the right-angled corners of a kite-shaped arena. Participants in group rectangle-kite were trained to find the hidden goal in one of the corners of a rectangle-shaped arena. Following this training, participants were given two 60-s test trials conducted in the absence of the hidden goal. For group rectangle-kite this test was in kite-shaped arena whereas, for group kite-rectangle, the test was in a rectangle-shaped arena. One test trial was conducted in an arena that had walls the same color as the arena in which participants were trained. If participants transfer the local shape-information from the training to the test arena then they should preferentially search in the corner(s) of the test arena that match the local geometric features of the training arena (Lew et al., 2014; McGregor et al., 2006; Pearce et al., 2004). A second test trial was conducted in which the test walls were a different color to the training walls. This test was designed to assess how susceptible to generalization decrement the transfer of local shape-information is; an effect that we were keen to avoid in Experiments 2 and 3.

### Method

#### Participants

Thirty-two participants were recruited from the University of Nottingham (26 female), and were given course credit toward the first year of their undergraduate psychology degree in return for participation. The age of participants ranged from 18- to 33-years-old (*M* = 21.72, *SD* = 5.00). A £10 prize was awarded to the participant who completed the experiment in the shortest time.

#### Materials

All virtual environments were constructed and displayed using Mazesuite software (Ayaz, Allen, Platek, & Onaral, 2008; www.mazesuite.com) which were run on a standard Stone desktop computer, running Microsoft Windows 7. A large Mitsubishi LDT422V LCD screen (935 mm × 527 mm) was used to display the virtual environments. All virtual arenas were viewed from a first-person perspective, and a grass texture was applied to the floor of each arena. Using the 0–255 RGB scale employed by Mazesuite, the cream colored walls used in the experiment were defined as 204, 178, 127, and the blue colored walls were defined as 178, 204, 229.

Assuming a walking speed similar to that in the real world (2 m/s), the perimeter of both the kite- and rectangle-shaped arenas was 72m (small walls: 9m, long walls: 27m). The height of the walls in both arenas was approximately 2.5 m. The kite-shaped arena contained two right-angled corners, and two corners with angles of 143.14° and 36.86°. The rectangular arena contained four right-angled corners. Finally, the goals within all arenas were square-shaped regions (1.08 m × 1.08 m, invisible to participants), the center of which was always located 2.48 m away from the walls of the arena, along on a notional line that bisected a right-angled corner in half.

A third arena was also used in this experiment, which was designed to allow participants to become familiar with the controls of the experimental task. This exploration arena was a regular octagon configured with red walls (RGB: 229, 25, 51), with a grass texture again applied to the floor. There was no hidden goal present. Again assuming a walking speed of 2 m/s, each wall of the exploration arena was 12 m in length.

#### Procedure

##### General

After signing a consent form, participants were given the following set of instructions on paper:

This study is assessing human navigation using a computer generated virtual environment. During this experiment, you will complete 20 trials. In each trial, you will be placed into a room that contains an invisible column. Your aim is to end the trials as quickly as possible by walking into the column.

You will view the environment from a first person perspective, and be able to walk into the column from any direction using the cursor keys on the keyboard. Once you’ve found the column a congratulatory message will be displayed and you should hit enter when you’re ready to begin the next trial. You will always be in the center of the arena when a trial begins, but the direction in which you face at the start of each trial will change.

To start with, you may find the column is difficult to find. The column does not move though, so it is possible to learn its specific location as the experiment goes along. It’s a good idea to fully explore the environment on the first few trials to become aware of your surroundings. This should help you in learning where the hidden column is.

This session should take around 15 minutes. If at any point you wish to stop this session, please notify the experimenter and you’ll be free to leave without having to give a reason why. Your results will be saved under an anonymous code, and kept confidential throughout.

The person who takes the least time to complete this experiment will win a £10 prize!

Participants sat not more than 100 cm from the screen, and were first provided with the opportunity to move around the octagonal exploration arena for two 30-s trials using the four keyboard cursor keys. Presses on the “up” and “down” cursor keys moved the participant forward and backward within the arena, respectively. Presses on the “left” and “right” cursor keys rotated the participant counterclockwise and clockwise within the arena, respectively. Following the exploration trials, participants completed the acquisition trials, in which they were required to find the hidden goal using the four cursor keys. These trials ended only when participants found the hidden goal, and once found, participants could no longer move, and a congratulatory message (*Congratulations, you found the goal!*) was displayed on screen. Participants pressed enter to begin the next trial. In the kite-shaped arena, participants always began each trial at a point halfway between the apex and obtuse corners. In the rectangle-shaped arena, participants began each trial in the center of the arena. The direction in which participants faced was randomized at the onset of each trial.

##### Acquisition

**Figure 1 fig1:**
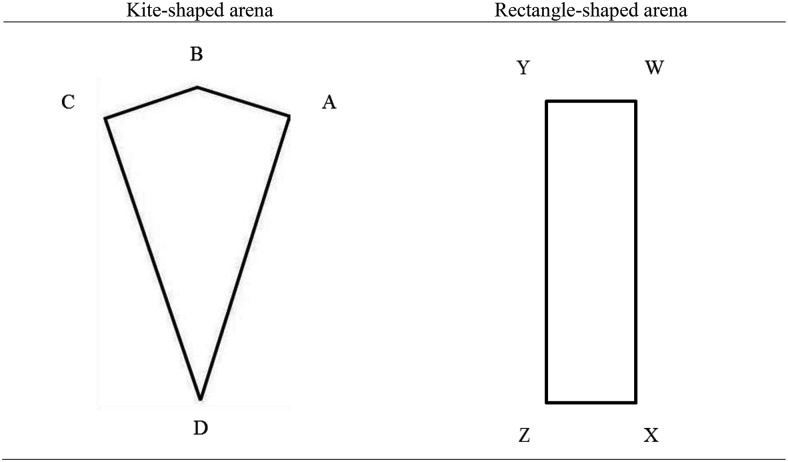
Schematic views of the kite- and rectangle-shaped arenas of Experiment 1. Letters are used to denote individual corners of each shape.

Sixteen participants received acquisition trials in a kite-shaped arena. For eight of these participants, the hidden goal was located in the right-angled corner where a short wall was to the left of a long wall (Corner A, Figure 1), whereas, for the other eight participants the goal was located where a long wall was to the left of a short wall (Corner C, Figure 1). When the goal was located in the corner where the short wall was to the left of the long wall, the whole arena was blue for four participants, and for the other four participants the arena was cream. This was also true for when the goal was in the corner where the long wall was to the left of a short wall. The remaining 16 participants received acquisition trials in a rectangle-shaped arena. The location of the hidden goal and the color of the walls, in the rectangle-shaped arena, were counterbalanced in the same manner as described for the kite-shaped arena. To ensure that visits to the correct corners of the rectangle were always rewarded, each rectangular arena contained two goal locations. When the goal was located in a right-angled corner where a short wall was to the left of a long wall, hidden goals were present in corners W and Z (see Figure 1). Similarly, when the goal was located where a long wall was to the left of a short wall, hidden goals were present in corners X and Y (see Figure 1).

##### Transfer tests

After 16 acquisition trials, participants immediately received two 60-s tests in which the hidden goal was removed. For participants who received acquisition trials in a kite-shaped arena, the transfer tests were conducted in rectangle-shaped arenas (group kite-rectangle) and, for participants who received acquisition trials in a rectangle-shaped arena, the transfer tests were conducted in kite-shaped arenas (group rectangle-kite). One transfer test was conducted in an arena that was the same color as the acquisition arena, and the other was conducted in an arena which was a different color to the acquisition arena. The order of the same- and different-color transfer tests was counterbalanced.

Performance during the transfer tests was analyzed using two methods. First, we measured the time spent in 3.24 m × 3.24 m square search zones that were placed at corners A and C of the kite-shaped arena (see Figure 1), and at all four corners of the rectangle-shaped arena. Assessing spatial behavior during extinction tests (where no hidden goal is present) in such a manner is common in both animal (McGregor, Horne, Esber, & Pearce, 2009), and human (Buckley, Smith, & Haselgrove, 2015a; Redhead & Hamilton, 2009) experiments. Second, following Pearce et al. (2004), we recorded which corner of the arena participants visited first during the test trials. A participant was deemed to enter a particular corner once they were within 3.24 m from the point where two walls joined.

### Results

In all experiments, we analyzed the data with analysis of variance (ANOVA). In addition, partial eta squared (η_p_^2^) was used to estimate effect sizes, and we report confidence intervals around this measure to depart from the dichotomous decisions offered by null-hypothesis significance testing. However, we wished to present coherent statistical data from both forms of analyses. In order to present confidence intervals around η_p_^2^ that are congruent with the outcomes of an ANOVA that uses .05 as the criterion for significance, we calculated 90% confidence intervals around η_p_^2^ (Steiger, 2004). Consequently, if the confidence interval surrounding η_p_^2^ excludes zero, the corresponding *p* value will indicate significance. Calculating 95% confidence intervals around η_p_^2^ can lead to cases where an *F* test returns a significant *p* value, but the confidence intervals for η_p_^2^ includes zero.

#### Acquisition

**Figure 2 fig2:**
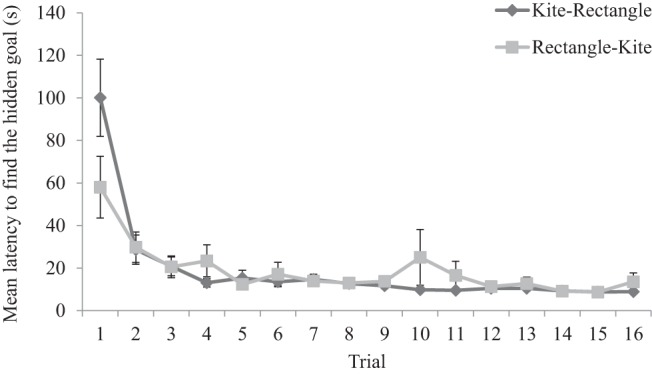
Mean latencies, for both the kite-rectangle and rectangle-kite groups, to find the hidden goal during the acquisition trials of Experiment 1. Errors bars represent ± one standard error of the mean.

Figure 2 shows that the latency, in seconds, from the beginning of each acquisition trial to enter the region defined as the hidden goal decreased during training in both kite- and rectangle-shaped arenas. A two-way ANOVA conducted on individual latencies to find the goal, with a between-subjects factor of group (kite-rectangle or rectangle-kite) and a within-subjects factor of trial (1–16), revealed no significant main effect of group, *F* < 1, but a significant main effect of trial, *F*(15, 450) = 19.04, *MSE* = 478.94, *p* < .001, η_p_^2^ = .39, 95% CI [.31, .42], and a significant interaction between group and trial, *F*(15, 450) = 2.46, *MSE* = 478.94, *p* = .002, η_p_^2^ = .08, 95% CI [.02, .09]. Participants in both groups became quicker to find the goal as trials progressed; however, participants trained in the kite-shaped arena were marginally slower to find the hidden goal on Trial 1 compared to participants trained in the rectangle-shaped arena, *F*(1, 30) = 3.28, *MSE* = 4318.34, *p* = .08, η_p_^2^ = .10, 95% CI [.00, .28]). There were no other significant differences between groups on remaining trials, *Fs*(1, 30) < 1.77, *MSE*s < 1369.39, *p*s > .19, η_p_^2^ < .06, 95% CI [.00, .22].[Fig-anchor fig2]

#### Transfer tests

##### Zone analysis

**Figure 3 fig3:**
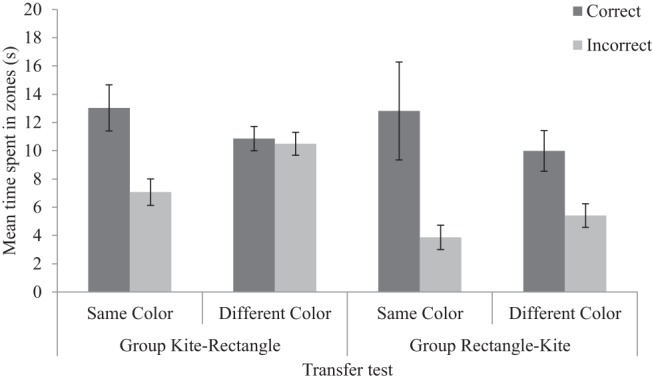
Mean time spent in the correct and incorrect zones, for both the kite-rectangle and rectangle-kite groups, during the same and different color transfer tests of Experiment 1. Errors bars represent ± one standard error of the mean.

Figure 3 displays the time, in seconds, that the kite-rectangle and rectangle-kite groups spent searching for the hidden goal, in both the correct and incorrect zones during the transfer tests. Correct zones were located at the right-angled corners of the test environment that shared the same local geometric cues as the corner that signaled the goal location during acquisition, and incorrect zones were located at the other right-angled corner(s). For participants in the rectangle-kite group, there was a clear preference for searching in correct zone, over the incorrect zone, during the same color transfer test. The same preference, albeit attenuated, was also apparent in the different-color transfer test for this group. Participants in the kite-rectangle group also preferentially searched in the correct zone over the incorrect zone, during the same color transfer test, but not on the different-colored test.[Fig-anchor fig3]

A three-way ANOVA conducted on individual time spent in zones, with a between-subjects factor of group (kite-rectangle or rectangle-kite), and within-subjects factors of test color (same or different) and zone (correct or incorrect), revealed no significant main effect of test color, *F* < 1, but a significant main effect of group, *F*(1, 30) = 4.32, *MSE* = 40.83, *p* = .046, η_p_^2^ = .13, 95% CI [.001, .31], indicating the kite-rectangle group spent significantly more time in the measured zones than the rectangle-kite group. There was also a significant main effect of zone, *F*(1, 30) = 14.46, *MSE* = 54.56, *p* = .001, η_p_^2^ = .33, 95% CI [.11, .50], as well as a significant interaction between zone and test color, *F*(1, 30) = 4.28, *MSE* = 54.56, *p* = .047, η_p_^2^ = .13, 95% CI [.0008, .31]. In both the same color test, *F*(1, 30) = 10.88, *p* = .003, η_p_^2^ = .27, 95% CI [.06, .44], and the different color test, *F*(1, 30) = 5.10, *p* = .031, η_p_^2^ = .15, 95% CI [.007, .33], participants searched for significantly longer in the correct zone compared to the incorrect zone. Across test colors, the amount of time participants spent in the correct zone did not significantly differ, *F*(1, 30) = 1.89, *p* = .18, η_p_^2^ = .06, 95% CI [.00, .22]; however, participants spent significantly longer in the incorrect zone during the different color test compared with the same color test, *F*(1, 30) = 6.49, *p* = .016, η_p_^2^ = .18, 95% CI [.02, .36]. Returning to the results of the overall ANOVA, the remaining interactions between zone and group, *F*(1, 30) = 1.90, *MSE* = 54.56, *p* = .18, η_p_^2^ = .06, 95% CI [.00, .22], between group and test color, *F* < 1, and the three-way interaction between zone, group, and test color, *F* < 1, were not significant.

##### First-choice analysis

Table 1 displays the number of participants in group rectangle-kite that first visited the correct, incorrect, acute, or obtuse corner of the same color, and different color, kite-shaped test arenas. Table 1 also shows the number of participants in group kite-rectangle that visited the correct, or incorrect, corners of the same color, and different color, rectangle-shaped test arenas. In both groups, for both colored tests, at least 75% of participants entered the correct corner first during the test trial.[Table-anchor tbl1]

Following Pearce et al. (2004), there are two possible navigational strategies that would lead participants to the correct corner of a test environment. First, according to a *local* strategy, participants navigate to the corner of the test arena that shared the same local geometric cues as the corner that signaled the goal location during acquisition. Second, according to a *single-wall* strategy, it is argued that participants navigate to one end of a particular wall during acquisition. For instance, if the goal was present in corners W and Z of the rectangle displayed in Figure 1, then participants could have learned to navigate to the left end of a long wall. If this behavior was transferred to the kite-shaped test environment, participants would be expected to navigate to the left end of wall AD (the correct corner), or the left end of wall CD (the acute corner). Alternatively, participants may have navigated to a particular end of a short wall. If the goal was present in corners W and Z of Figure 1, then participants could learn to navigate to the right end of a short wall. If this behavior was transferred to the kite-shaped test arena, participants would navigate either to the left end of wall AB (the correct corner) or the left end of wall CB (the obtuse corner).

By analyzing the first-choice behavior of group rectangle-kite, it is possible to determine which strategy participants were using in the current experiment by process of elimination. If participants were using a *local* strategy, it would be expected that there would be significantly more first visits to the correct corner, over any other corner, at test. In contrast, the *single-wall* strategy predicts that participants will visit the correct corner first on only half of the test trials. For the other half of the test trials, participants would be expected to visit either the acute or obtuse corner, depending on whether they used a long- or short-wall strategy. By following a *single-wall* strategy, the probability of choosing the correct corner, over both the acute and obtuse corners combined, is .5. Across both test trials given to group kite-rectangle, the correct corner was visited first on 24 out of 32 occasions. A sign test revealed that this outcome was significantly greater than chance (*p* = 0.007), thus, providing evidence that participants were not using a *single-wall* strategy. For the sake of completeness, across both tests administered to group kite-rectangle, participants navigated to the correct corner first on 26 out of 32 occasions. A sign test revealed that this outcome was also significantly different to chance (*p* < 0.001).

### Discussion

Following training in which participants were required to find a goal hidden in one of the right-angled corners of either a kite- or rectangle-shaped environment, participants were transferred to a rectangle- or kite-shaped testing environment, respectively. Within these test environments, participants spent more time exploring the corner that had the same geometric cues of the corner that was closest to the goal in the training environment. Moreover, participants displayed a significant preference for navigating to the correct corner first during a test trial. Together, these results are consistent with other experiments that have demonstrated similar navigational transfer effects across environments of different shapes (Lew et al., 2014; McGregor et al., 2006; Pearce et al., 2004; Tommasi & Polli, 2004), and are consistent with the idea that, during training, participants used a local geometric-cue in order to find the hidden goal. For example, participants may have learned during training that approaching an egocentrically encoded scene, such as the conjunction of two walls of different lengths, was associated with the goal (Cheung, Stürzl, Zeil, & Cheng, 2008; McGregor et al., 2006; Pearce et al., 2004; Stürzl, Cheung, Cheng, & Zeil, 2008). As the same, or a similar, scene is present during the test trials, this navigational behavior will transfer from training. It is rather more difficult to explain these results in terms of a theory of spatial navigation that proposes a global representation of the overall shape of the environment is learned during training (Cheng, 1986; Gallistel, 1990; Wang & Spelke, 2002). If this were the case, then the change in the overall shape of the environment between training and testing should have removed any preference for searching in one right-angled corner over another.

It is worthwhile discussing the interaction between group and trial that was observed during acquisition, and the main effect of group that was observed at test. These effects were most likely observed because, compared with the kite-shaped arena, the rectangle-shaped arena had twice as many goals and zones during acquisition and test, respectively. Consequently participants will be more likely to find the goal by chance in the rectangle-shaped environment that contained two hidden goals, compared with the kite-shaped arena that contained one hidden goal on Trial 1. Similarly, at test, there were two correct and incorrect zones in the rectangle-shaped arena, compared with one of each zone in the kite-shaped arena. Again, therefore, it would be expected that participants who were tested in a rectangle-shaped environment would spent more time in zones, overall, than participants tested in a kite-shaped environment.

Figure 3 shows that, when the color of the training and test environments differed, the transfer of navigational behavior from a rectangle to a kite was, at least numerically, less susceptible to generalization decrement relative to the transfer of navigational behavior from a kite to a rectangle, although the lack of a three-way interaction fails to confirm this finding. Experiments 2 and 3 were designed to assess the extent to which local geometric information could be blocked by, and block, learning about wall color information, respectively. In order to achieve this, it was necessary to transfer participants to different colored arenas. In order to protect the effects we observed from being confounded by generalization decrement, we ensured that this transfer was from a rectangle to a kite, and not vice versa.

As we noted in the introduction, the model proposed by Miller and Shettleworth (2007, 2008, 2013) provides an explanation for the presence, and absence, of cue competition effects in the spatial domain. It does not, however, state how organisms encode geometric information. Experiment 1 (see also: Lew et al., 2014; McGregor, Jones, Good, & Pearce, 2006; Poulter, Kosaki, Easton, & McGregor, 2013; Tommasi & Polli, 2004) shows that navigation that is based upon local geometric information transfers to an environment that has a different overall shape. Experiment 2, therefore, used this transfer procedure to determine if learning about local geometric cues could be blocked by learning about wall color information.

## Experiment 2

**Figure 4 fig4:**
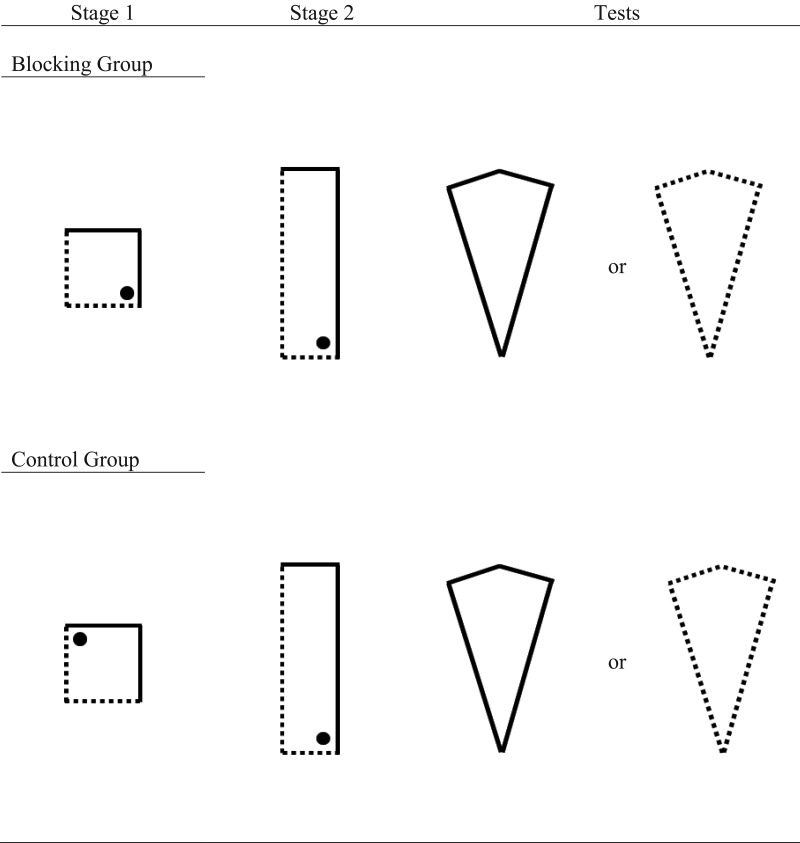
An example of the trials given to the blocking and control groups during Experiment 2. The dotted and solid lines represent different colored walls, and the black filled circles represent the location of the hidden goal. Participants received one test trial in a kite-shaped arena, the color of which was counterbalanced across participants.

The current experiment was designed to assess if learning about local geometric information is subject to blocking from prior learning about non-geometric wall colors. Evidence that appears consistent with this prediction was reviewed in the introduction to this article (e.g., Pearce et al., 2006); however, in all previous demonstrations of blocking, both in rats (e.g., Horne & Pearce, 2009) and humans (e.g., Wilson & Alexander, 2008), test trials have been conducted in the same shaped arena that was used for Stage 2 training. It is, thus, not possible to determine whether these experiments have detected blocking of *global* geometric learning or, instead, blocking of *local* geometric learning. In Experiment 2, we adapted the design of Pearce et al. (2006) so that the arenas at test contained the same local geometric-cues as the arena used during Stage 2 training but, importantly, the global shape of these two environments were different. Any difference in test trial performance in the blocking and control groups would, therefore, be a consequence of participants navigating on the basis of local shape-information, and not a representation of the global shape. In Stage 1, participants were trained to find a hidden goal in a square-shaped arena that comprised two adjacent blue walls and two adjacent cream walls. For a blocking group, the goal was located in a corner where, for example, a blue wall was to the right of a cream wall. For a control group, the goal was located in a corner where, for example, a blue wall was to the left of a cream wall. In Stage 2, participants were transferred to a rectangle-shaped environment that also comprised two adjacent blue walls and two adjacent cream walls. For both groups, the hidden goal was located, for example, in the corner where a long blue-wall was to the right of a short cream-wall. Consequently, participants in both groups could rely on the shape of the environment to find the hidden goal, or the color of the walls. For the blocking group, however, the color of the walls that signaled the goal location in Stage 1 continued to signal the goal location in Stage 2. In contrast, for the control group, the color of walls that signaled the goal location in Stage 1 no longer signaled the goal location in Stage 2 (see Figure 4).[Fig-anchor fig4]

Following training, participants were given two 60-s test trials in a kite-shaped arena that contained no hidden goals. If learning about local geometric cues proceeds in a manner consistent with the theory proposed by Miller and Shettleworth (2007, 2008, 2013), the associative strength of the colored walls in the goal location should prevent the blocking group learning the association between the local geometric cues and the goal location in Stage 2 of the experiment. This group should, therefore, show no preference for any corner of the kite-shaped arena in the final test trials. For the control group, the associative strength of the colored walls in the goal location during Stage 2 will initially be low because, in Stage 1, this wall color did not signal the goal location. Consequently, the theory proposed by Miller and Shettleworth (2007, 2008, 2013) predicts that the local shape-information may enter into an association with the hidden goal. Participants in the control group, therefore, should show a preference for the corner of the kite that shares the same local geometric cues as the corner of the rectangle that signaled the goal location in Stage 2.

### Method

#### Participants

Thirty-two participants were recruited from the University of Nottingham (20 female), and were given course credit toward the first year of their undergraduate psychology degree in return for participation. The age of participants ranged from 18- to 46-years-old (*M* = 22.81, *SD* = 5.34). Participants were pseudorandomly assigned to each group in order to ensure an equal number of males and females were allocated to the blocking and control groups. A £10 prize was awarded to the participant who completed the experiment in the shortest time.

#### Materials

All virtual environments were created and displayed as described in Experiment 1. The cream- and blue-colored walls that are referred to in the following procedure section are the same as described in Experiment 1. However, in Experiment 2 a square-shaped arena was employed in Stage 1. Assuming a walking speed of 2 m/s, the perimeter of the square was 72 m (each wall: 18 m). The height of the walls creating the square was, again, approximately 2.5 m. As with the kite- and rectangle-shaped arenas, the goal within the square-shaped arena was a square region (1.08 m × 1.08 m, invisible to participants), the center of which was always located 2.48 m away from the walls of the arena, along on a notional line that bisected a right-angled corner in half.

#### Procedure

##### General

All general details were the same as reported for Experiment 1, save for minor changes to the instructions. Participants were informed that there would be 30 trials in the experiment, and that the session would last around 20 minutes.

##### Stage 1

Participants first completed 16 trials in a square-shaped arena, which comprised two adjacent cream walls, and two adjacent blue walls. Participants began each trial at the center of the arena, facing a randomly selected direction on every trial. The hidden goal, for both blocking and control groups, was located in a corner where two differently colored walls met. For half of the participants in both the blocking and the control groups the goal was hidden in a corner where a cream wall was to the left of a blue wall; for the remaining participants, the goal was located in the corner where a blue wall was to the left of a cream wall.

##### Stage 2

Immediately after completing Stage 1, participants completed 12 trials in a rectangle-shaped arena. The rectangle-shaped arena in Stage 2 comprised two adjacent cream walls, and two adjacent blue walls. For the blocking group, the colored walls that previously predicted the goal location in the square shaped arena of Stage 1 continued to predict the goal location in the rectangle-shaped arena in Stage 2. For the control group, however, the colored walls that previously signaled the goal location in Stage 1 no longer signaled the goal location in Stage 2. Instead, the goal was located at the corner of the rectangular shaped arena that was a mirror image of the colored walls that signaled the goal location in Stage 1. For example, if the goal was located in a corner where a cream wall was to the left of a blue wall in Stage 1, then the goal would be located in a corner where a blue wall was to the left of a cream wall in Stage 2. The color of the walls forming the rectangle-shaped arena was fully counterbalanced with the positioning of the goal within the arena.

##### Test trials

Participants received two test trials, each of which contained no hidden goal. For each test, participants were allowed to search for 60 s in a kite-shaped arena. The first test trial was administered after participants had completed four trials of Stage 2, while the second test trial was administered after participants had completed 12 trials of Stage 2. Each participant received two tests with arenas that were the same color—for half of the participants this was blue, for the remaining participants this was cream. As described for Experiment 1, the time spent within search zones were used to measure navigational performance during these test trials.

### Results

#### Stage 1

**Figure 5 fig5:**
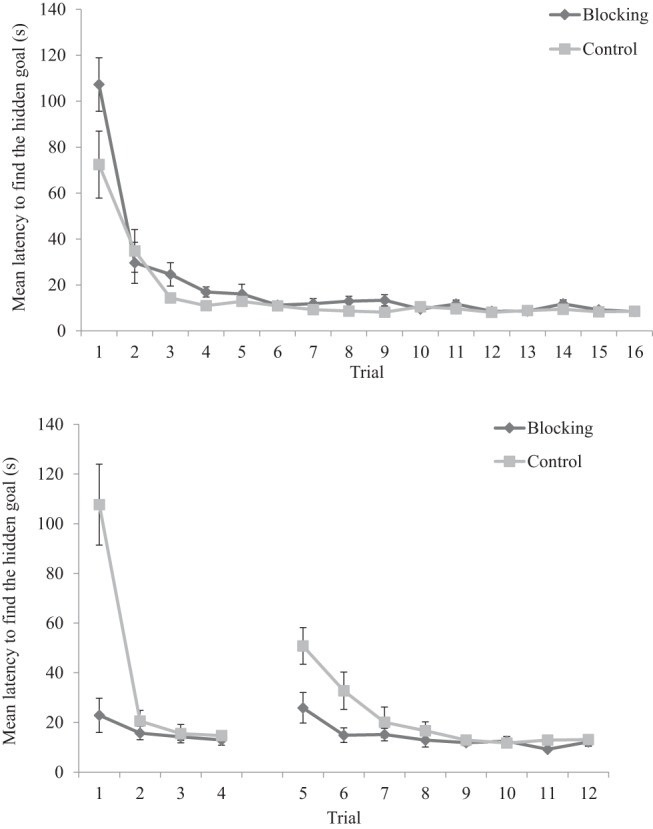
Mean latencies, for both the blocking and control groups, to find the hidden goal during Stage 1 (upper panel) and Stage 2 (lower panel) of Experiment 2. Errors bars represent ± one standard error of the mean.

The upper panel of Figure 5 shows the latency, in seconds, from the beginning of each trial to enter the region defined as the hidden goal for the blocking, and control groups. Both groups displayed a reduction in latencies across the early training trials. A two-way ANOVA conducted on individual latencies to find the goal, with a between-subjects factor of group (blocking or control) and a within-subjects factor of trial (1–16), revealed a significant main effect of trial, *F*(15, 450) = 45.29, *MSE* = 288.84, *p* < .001, η_p_^2^ = .60, 95% CI [.54, .63], confirming that participants became quicker to find the goal as training progressed. There was no main effect of group, *F*(1, 30) = 3.85, *MSE* = 563.25, *p* = .06, η_p_^2^ = .11, 95% CI [.00, .29]; however, the interaction between trial and group was significant, *F*(15, 450) = 2.20, *MSE* = 288.84, *p* = .006, η_p_^2^ = .07, 95% CI [.01, .07]. Simple main effects analysis revealed that the control group found the goal faster only on Trials 4 and 9, *F*s(1, 30) > 4.28, *p*s < .047, η_p_^2^ = .13, 95% CI [.0008, .31].[Fig-anchor fig5]

#### Stage 2

The lower panel of Figure 5 shows the latency, in seconds, from the beginning of each trial to enter the region defined as the hidden goal for both the blocking and control groups. Mean latencies to find the goal were quicker in the blocking group, compared with the control group, on Trials 1, 5, and 6 but there was little indication of any difference between the groups during trials immediately before the administration of the two test trials. A two-way ANOVA conducted on individual latencies to find the goal, with a between-subjects factor of group (blocking or control) and a within-subjects factor of trial (1–12), revealed a significant main effect of trial, *F*(11, 330) = 24.35, *MSE* = 327.26, *p* < .001, η_p_^2^ = .45, 95% CI [.37, .49], of group, *F*(1, 30) = 11.82, *MSE* = 1253.92, *p* = .002, η_p_^2^ = .28, 95% CI [.19, .32], and a significant interaction between trial and group, *F*(11, 330) = 14.14, *MSE* = 327.26, *p* < .001, η_p_^2^ = .32, 95% CI [.23, .36]. Simple main effects analysis revealed that the blocking group were significantly faster to find the goal on Trials 1, 5, and 6, *F*s(1, 30) > 4.92, *p*s< .034, η_p_^2^ > .14, 95% CI [.01, .32].

#### Test trials

**Figure 6 fig6:**
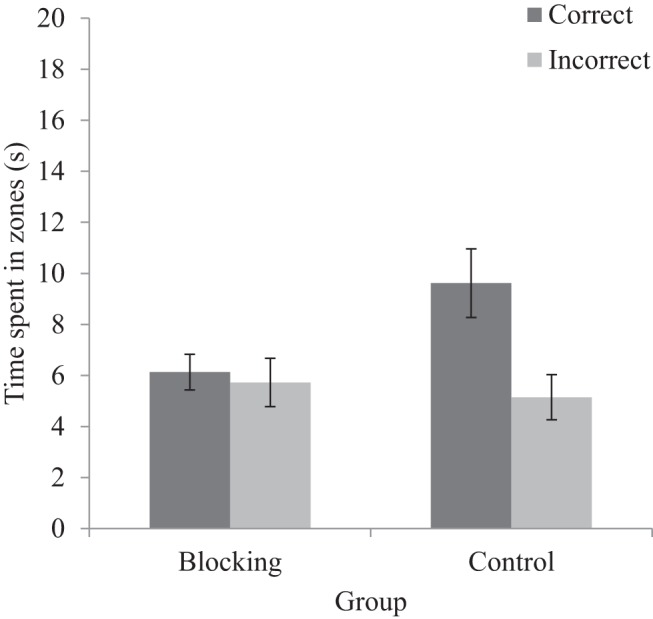
Mean time spent, for both the blocking and control groups, in the correct and incorrect zones during the test trial of Experiment 2. Errors bars represent ± one standard error of the mean.

Figure 6 shows the amount of time that participants spent in both the correct and incorrect zones averaged across the two test trials. The correct zone was defined as the corner of the kite-shaped arena that shared the same local geometry as the corner of the rectangular-shaped arena that contained the hidden goal in Stage 2. The incorrect zone was defined as the other right-angled corner. The blocking group spent no more time searching for the goal in the correct than the incorrect zone, whereas the control group spent more time in the correct, than the incorrect, zone. A three-way ANOVA conducted on individual time spent in zones, with a between-subjects factor of group (blocking or control), and within-subjects factors of zone (correct or incorrect) and test (first or second), revealed no significant main effect of group, *F*(1, 30) = 1.77, *MSE* = 38.35, *p* = .19, η_p_^2^ = .06, 95% CI [.00, .22], or test, *F* < 1. There was, however, a significant main effect of zone, *F*(1, 30) = 7.61, *MSE* = 25.08, *p* = .010, η_p_^2^ = .20, 95% CI [.03, .39], and a significant interaction between zone and group, *F*(1, 30) = 5.26, *MSE* = 25.08, *p* = .029 η_p_^2^ = .15, 95% CI [.01, .33]. Simple main effects analysis of this interaction revealed that the control group spent more time searching in the correct zone than the blocking group, *F*(1, 30) = 5.33, *p* = .028, η_p_^2^ = .15, 95% CI [.01, .33]. There was, however, no difference in the time spent in the incorrect zone between the blocking and control groups, *F* < 1. Within groups, the blocking group did not spend more time in the correct zone than the incorrect zone, *F* < 1, whereas the control group did spend more time in the correct zone, compared to the incorrect zone, *F*(1, 30) = 12.76, *p* = .001, η_p_^2^ = .30, 95% CI [.09, .47]. The remaining two way interactions between test and group, test and zone, and the three-way interaction were not significant, *F*s < 1.[Fig-anchor fig6]

### Discussion

Participants received training in which a hidden goal was located in a distinctively colored corner of a rectangle, before receiving test trials in a kite-shaped arena. For a blocking group, the distinctively colored corner had previously been established as a cue for the hidden goal, and this pre-training resulted in participants spending no more time in the correct than the incorrect zone during the final test stage. In contrast, for the control group, the distinctively colored corner had not been previously established as a cue for the hidden goal, and this pre-training resulted in the control group spending more time in the correct than the incorrect zone at test. These data, therefore, constitute a demonstration of blocking, and concord with those obtained by Pearce et al. (2006), who also demonstrated that establishing a wall color as a cue for a goal location could block subsequent learning about the location of a hidden goal with respect to the shape of the arena. For the current experiment, however, the shape of the arena was changed between Stage 2 and testing; thus, the learned information that permits navigation to transfer between arenas of different shapes is susceptible to blocking. The current experiment, therefore, suggests that learning about local geometric cues is consistent with the predictions provided by the Miller-Shettleworth model, which suggests that navigation that is based upon the shape of an environment is a consequence of an associative process.

The model of navigation proposed by Miller and Shettleworth (2007, 2008, 2013) proposes that learning to navigate on the basis of non-geometric information (e.g., colored walls) is governed by the same principles as learning to navigate on the basis of the boundary shape of an environment (cf. Cheng, 1986; Gallistel, 1990). Consequently, if learning about local geometric information proceeds in a manner consistent with the Miller-Shettleworth model, then learning about colored-wall information should not only block learning about local geometric cues, but also be subject to blocking by local geometric cues. Experiment 3 was conducted to test this prediction.

## Experiment 3

The current experiment was designed to assess if learning about local shape-information would block subsequent learning about wall-color information. In order to do this, the design of Experiment 2 was altered so that participants were first placed into a uniformly colored rectangle-shaped arena and required to find a hidden goal in, for example, the corner where a long wall was to the left of a short wall. In Stage 2, participants were transferred to a kite-shaped arena in which the two long walls were a different color to the two short walls. For the blocking group, the hidden goal was located in a right-angled corner where a long wall was to the left of a short wall. As this corner shares the same local-shape features as the corner that contained the hidden goal in Stage 1, we do not expect participants to acquire any knowledge about the colored walls that also predict the goal location in Stage 2. For a control group, the goal was located in the right angled corner of the kite where a long wall was to the right of a short wall in Stage 2. As the local shape properties of this corner were not paired with the hidden goal in Stage 1 for the control group, participants should associate the wall color with the goal location in this stage. In a final test, participants were given a trial in a square-shaped arena constructed from the same wall colors present in the kite-shaped arena. We expected participants in the control group, but not the blocking group, to preferentially search in the corner of the square that shared the same color configuration as the location that contained the hidden goal in the kite-shaped arena from Stage 2. The blocking group should show no preference for any corner of the square test arena.

It might be expected that the control group would learn less about the local geometric cues that signal the goal location, compared with the blocking group. In order to assess this possibility, we compared learning with the geometric properties of the Stage 2 arena in the control group, to that of the blocking group, by including a test trial in a kite-shaped arena that was built from gray walls. Furthermore, as there was no effect of test in Experiment 2, in the current experiment we administered only one set of tests following four trials of Stage 2 training.

### Method

#### Participants

Forty-eight participants were recruited from the University of Nottingham (36 female), and were given course credit toward the first year of their undergraduate psychology degree in return for participation. The age of participants ranged from 18 to 41 years (*M* = 19.92, *SD* = 3.93). Participants were pseudorandomly assigned to each group in order to ensure an equal number of males and females were allocated to each group. A £10 prize was awarded to the participant who completed the experiment in the shortest time.

#### Materials

The dimensions of the kite- and rectangle-shaped arenas were the same as reported for Experiment 2. The square arena had a perimeter of 54m (each wall: 13.5 m). A number of experiments have observed an attenuation, or a complete absence, of blocking when the to-be-blocked cue is of a higher salience than the blocking cue (e.g., Denniston, Miller, & Matute, 1996; Denton & Kruschke, 2006; Hall, Mackintosh, Goodall, & Dal Martello, 1977; Miller & Matute, 1996). In order to protect the present experiment from this effect, we reduced the salience of the wall colors, relative to Experiment 2, by making the two different wall colors subtly different shades of pink (RGB: 178, 76, 204) and purple (RGB: 153, 0, 204).

#### Procedure

##### General

All general details were the same as reported for Experiment 1, save for minor changes to the instructions. Participants were informed that there would be 22 trials in the experiment.

##### Stage 1

**Figure 7 fig7:**
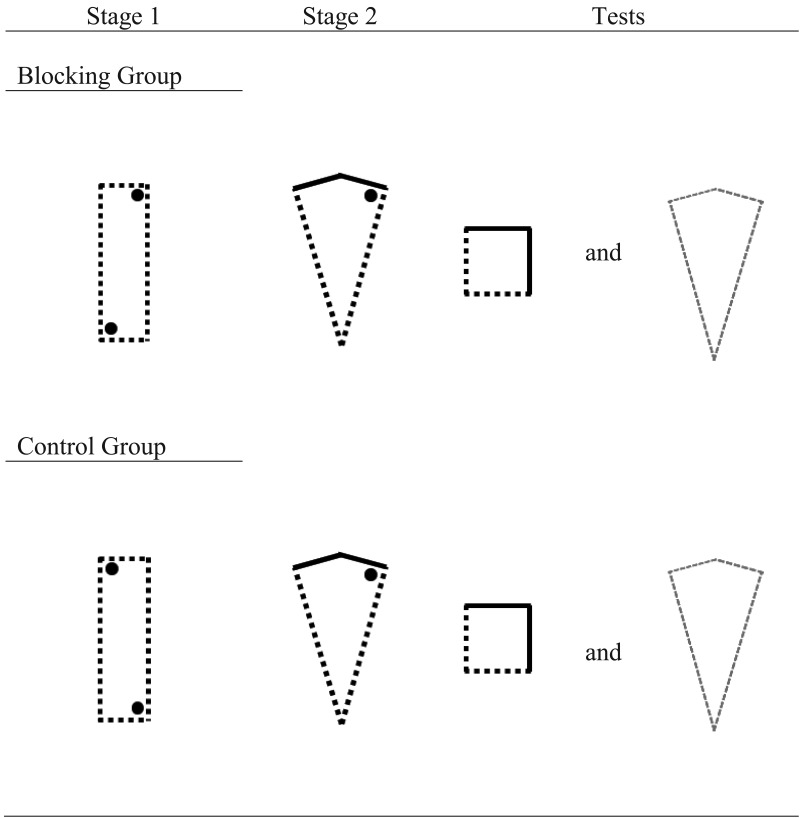
An example of the trials given to the blocking and control groups during Experiment 3. The dotted and solid lines represent different colored walls, and the black filled circles represent the location of the hidden goal. Participants received two test trials, one in a square-shaped environment and one in a kite-shaped arena.

Participants were first given 16 trials in a rectangle-shaped arena, the walls of which were either all pink, or all purple, in color. For half the participants, the hidden goal was located in a corner where a short wall was the left of a long wall, whereas, for the other half of the participants, the goal was located in a corner where a long wall was to the left of a short wall. As with Experiment 1, to ensure visits to the correct corner of the rectangle were always rewarded, each rectangle-shaped arena contained two hidden goals (see Figure 7). Each goal location was used equally often in each differently colored arena, and each group was trained to find the goal in each corner an equal number of times.[Fig-anchor fig7]

##### Stage 2

Following Stage 1, participants immediately completed four trials in a kite-shaped arena which comprised two pink and two purple walls. In the blocking group, the hidden goal was located in the corner of the kite that shared the same local geometric features that signaled the goal location in Stage 1. Consequently, if the hidden goal was located in a corner where the short wall was the left of a long wall in the rectangle-shaped arena during Stage 1 training, then the goal would be located in the corner where the short wall was the left of a long wall in the kite-shaped during Stage 2. For half the participants in the blocking group, the long walls of the kite were purple and the short walls were pink whereas, for the other half of participants, the long walls were pink and the short walls were purple. The color of the walls was counterbalanced in the same manner as for participants in the control group. However, in the control group the hidden goal was located in the corner of the kite that shared the same local geometric features that signaled the absence of the goal in Stage 1.

##### Test trials

After completing Stage 2, participants were given two test trials, both of which lasted for 60 s, and both of which contained no hidden goal. In the color test, participants were placed into a square arena that consisted of two adjacent pink walls, and two adjacent purple walls. In the shape test, participants were placed into a kite-shaped arena which consisted of four gray walls. The order in which these two tests were administered was counterbalanced across participants. Navigational behavior in the kite-shaped arena was measured as described for the previous experiments reported here. Behavior in the square-shaped arena was measured in a similar manner; however, as the square arena was smaller than the kite-shaped arena, we reduced the area of the zones accordingly. We, therefore, measured the time spent in square shaped zones (2.16 m × 2.16 m) located at the each corner of the square arena. The center of each zone was located 2.48 m from the corners of the arena, along a line that bisects the corner in half.

### Results

#### Stage 1

**Figure 8 fig8:**
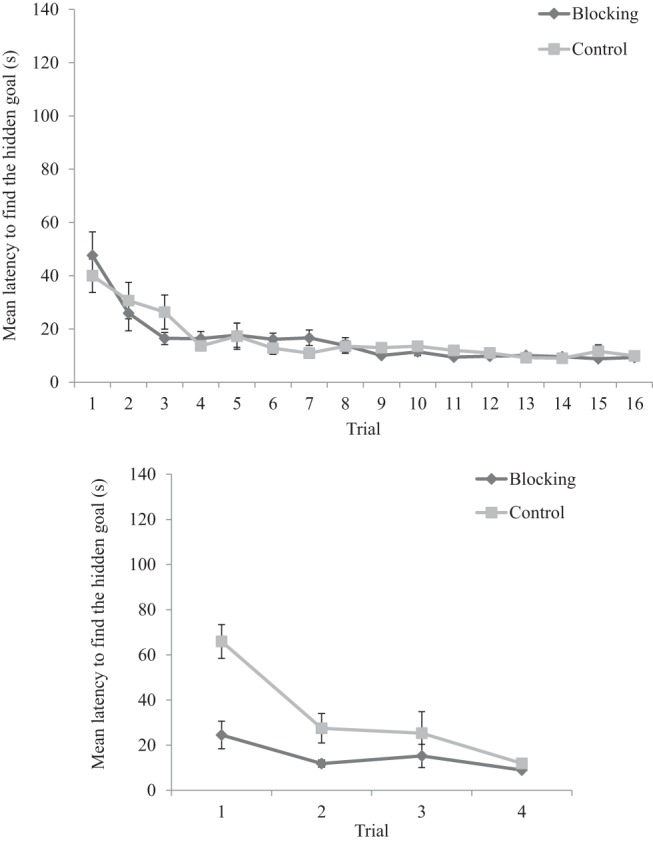
Mean latencies, for both the blocking and control groups, to find the hidden goal during Stage 1 (upper panel) and Stage 2 (lower panel) of Experiment 3. Errors bars represent ± one standard error of the mean.

The upper panel of Figure 8 shows the latency, in seconds, from the beginning of each trial to enter the region defined as the hidden goal, for both the blocking and control groups. Mean latencies decreased across the early trials of Stage 1 but there was little evidence of any between-groups differences in the latter stages of Stage 1. A two-way ANOVA of individual latencies to find the goal, with a between-subjects factor of group (blocking or control) and a within-subjects factor of trial (1–16), revealed a significant main effect of trial, *F*(15, 690) = 16.12, *MSE* = 242.26, *p* < .001, η_p_^2^ = .26, 95% CI [.20, .29], confirming that participants took less time to find the hidden goal as Stage 1 training progressed. There was, however, no significant main effect of group, and no significant interaction between group and trial, *F*s < 1.[Fig-anchor fig8]

#### Stage 2

The lower panel of Figure 8 shows the latency, in seconds, from the beginning of each trial to enter the region defined as the hidden goal, for both blocking and control groups during Stage 2. Mean latencies for the blocking group were quicker than the control group on Trials 1 and 2, although the performance of the two groups appeared more closely matched on Trials 3 and 4. A two-way ANOVA of individual latencies to find the goal, with a between-subjects factor of group (blocking or control) and a within-subjects factor of trial (1–4), revealed significant main effects of trial, *F*(3, 138) = 15.67, *MSE* = 681.63, *p* < .001, η_p_^2^ = .25, 95% CI [.14, .34], group, *F*(1, 46) = 14.44, *MSE* = 1021.91, *p* < .001, η_p_^2^ = .24, 95% CI [.13, .32], and a significant interaction between group and trial, *F*(3, 138) = 4.94, *MSE* = 681.63, *p* = .003, η_p_^2^ = .10, 95% CI [.02, .17]. Simple main effects analysis revealed that the blocking group found the hidden goal quicker than the control group on Trials 1, 2, and 4, *F*s(1, 46) < 18.43, *MSEs* < 1117.64, *p*s< .024, η_p_^2^ > .11, 95% CI [.01, .25].

#### Test trials

##### Color test

**Figure 9 fig9:**
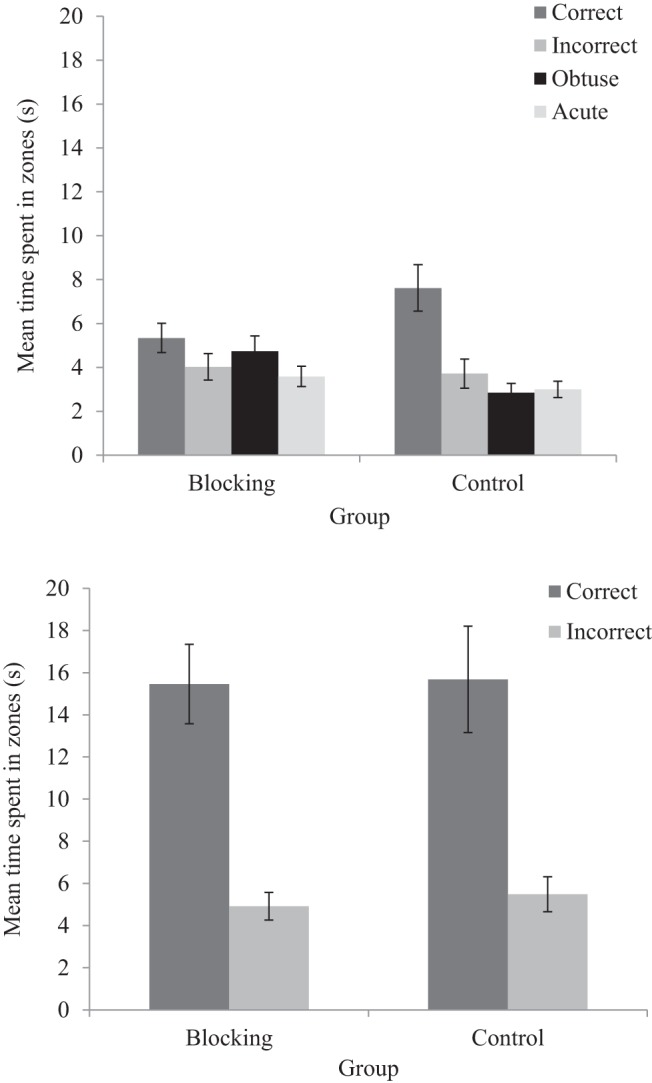
Mean time spent, for both the blocking and control groups, in the correct and incorrect zones during the color test (upper panel) and shape test (lower panel) of Experiment 3. Errors bars represent ± one standard error of the mean.

The upper panel of Figure 9 shows the amount of time, in seconds, participants spent searching for the hidden goal in all four zones of the square-shaped arena. The wall colors at each corner of the square were unique, and were the same as those that were present in the kite-shaped arena in which participants navigated during Stage 2. Consequently, we identify each corner of the square with reference to the corners that this color occupied in Stage 2. Participants in the blocking group spent an equivalent amount of time in each of the four search zones. In contrast, participants in the control group showed a preference for searching in the correct zone of the arena, relative to the remaining three zones. A two-way ANOVA conducted on individual time spent in zones, with a between-subjects factor of group (blocking or control), and a within-subjects factors of zone (correct, incorrect, obtuse, or acute), revealed no significant main effect of group, *F* < 1. There was, however, a significant main effect of zone, *F*(3, 138) = 9.18, *MSE* = 10.76, *p* < .001, η_p_^2^ = .17, 95% CI [.07, .25], and a significant interaction between zone and group, *F*(3, 138) = 3.40, *MSE* = 10.76, *p* = .02, η_p_^2^ = .07, 95% CI [.01, .13]. Simple main effects analysis showed that participants in the control group preferentially searched in the correct zone over all other zones, *F*(3, 44) = 7.21, *p* < .001, η_p_^2^ = .33, 95% CI [.11, .45]. In contrast, participants in the blocking group did not spend significantly longer in any of four zones, *F*(3, 44) = 1.39, *p* = .26, η_p_^2^ = .09, 95% CI [.00, .19].[Fig-anchor fig9]

##### Shape test

The lower panel of Figure 9 shows the amount of time, in seconds, participants spent searching for the hidden goal in both the correct and incorrect zones of the kite-shaped arena. The correct zone was located at the right angled corner that signaled the goal location during Stage 2 training, and the incorrect zone was located at the other right angled corner. Both the blocking and control groups spent more time searching in the correct zone than the incorrect zone. A two-way ANOVA conducted on individual time spent in zones, with a between-subjects factor of group (blocking or control), and within-subjects factors of zone (correct or incorrect), revealed only a significant main effect of zone, *F*(1, 46) = 35.13, *MSE* = 73.46, *p* < .001, η_p_^2^ = .43, 95% CI [.25, .56], confirming that participants spent more time in the correct zone relative to the incorrect zone. There was no significant main effect of group, or a significant interaction between group and zone, *F*s < 1.

### Discussion

Participants received training in Stage 1 in which a hidden goal was located in one of the right-angled corners of a rectangle-shaped arena. Following this training, participants were required to find the hidden goal in a kite-shaped arena, the walls of which were distinctive colors. For the blocking group, the hidden goal remained in the same right-angled corner as during training. For the control group, the hidden goal was placed in the other right-angled corner. Following this training test trials were administered in a square arena that comprised walls of the same color as the arena from Stage 2. The results of this test revealed that participants in the control, but not the blocking, group spent longer searching in the corner whose color was the same as that rewarded during Stage 2. The blocking group showed no such preference. This result complements the results of Experiment 2, demonstrating that the geometric features that permit navigation to transfer between arenas of different shapes is able to prevent (block) learning about the wall color of the arena.

Interestingly, participants in both groups displayed an equal, and strong, preference for searching in the correct, over the incorrect, corner during a test in which they were placed into a uniformly colored kite-shaped arena. The blocking group had been consistently rewarded for navigating to the same corner throughout Stages 1 and 2; thus, a strong preference for the correct corner was expected in this group. In the control group, however, participants were first trained to navigate to a corner where, for example, a short wall was to the left of a long wall in Stage 1, and then trained to navigate to a corner where a long wall was to the left of a short wall in Stage 2. Given this inconsistent training, a strong preference for the correct corner in the control group was somewhat surprising. The Miller-Shettleworth model, however, can accommodate this finding because it incorporates a choice rule into the Rescorla-Wagner learning algorithm. If the correct geometry had lower absolute associative strength in the control group, relative to the blocking group, the model can still predict equal performance from both groups so long as the correct corner has higher associative strength than the incorrect corner. For the associative strength of the correct corner in Stage 2 to have more associative strength than the incorrect corner in the control group, it is necessary for Stage 2 training to reverse the strength of the associative links formed in Stage 1. This would be possible if (a) due to generalization decrement (Blough, 1975), the associative strength gained by cues in Stage 1 did not transfer completely to Stage 2; and/or (b) the local geometric cues were more salient than the wall color information, something that is entirely plausible given that we chose to conduct the experiment with low salience wall colors.

## General Discussion

In Experiment 1, participants who were trained in a virtual arena to locate a hidden goal in one corner of, for example, a rectangle-shaped arena subsequently expressed a bias toward searching in a corner of the same local geometry that was in an arena of a different global shape. These results are consistent with comparable experiments conducted with rats in aquatic and dry arenas (e.g., McGregor et al., 2006; Pearce et al., 2004; Tommasi & Polli, 2004), and studies of navigation in adults in virtual worlds (e.g., Lew et al., 2014). This general effect, in which spatial navigation that is based upon an environment’s geometry survives a transformation of its overall shape, has been interpreted as evidence of an encoding of the local geometric features of an environment during navigation (Pearce et al., 2004; McGregor et al., 2006; Tommasi & Polli, 2004), and the first-choice data from our Experiment 1 support this conclusion. This interpretation contrasts with alternative conceptions of spatial navigation based upon the global shape of the environment, (e.g., Cheng, 1986; Gallistel, 1990). In Experiment 2, learning the location of a hidden goal with respect to non-geometric wall colors in a square arena blocked subsequent learning about the goal’s location with respect to the local geometric information. Experiment 3 demonstrated that this blocking effect was reciprocal. Learning to locate the hidden goal with respect to the geometric cues blocked subsequent learning about colored walls. Together, the results of Experiments 2 and 3 suggest that local geometric cues are permitted to compete with non-geometric cues, for associative strength to a goal location, according to the rules proposed by Miller and Shettleworth (2007, 2008, 2013).

The results of Experiment 2 are consistent with previously reported experiments where non-geometric cues have blocked learning about geometric cues (e.g., Horne & Pearce, 2009; Pearce et al., 2006; Wilson & Alexander, 2008). In these experiments, however, test trials were conducted in an arena that was of the same overall shape as the arena used in Stage 2 training. Consequently it is not possible to distinguish whether learning about wall colors had blocked learning about a representation of *global,* or *local,* geometric cues. Similarly, the results of Experiment 3 are consistent with previous reports of navigation based upon shape-information blocking learning about navigation based upon landmarks (e.g., Wilson & Alexander, 2008). Again, however, as an arena of the same overall shape was employed in Stages 1 and 2 of this experiment, it is not possible to determine whether learning about *global* or *local* geometric cues blocked subsequent learning about non-geometric cues. Where our experiments distinguish themselves is through the change of shape between Stage 2 and Test (Experiment 2), or between Stage 1 and Stage 2 (Experiment 3). This manipulation allowed us to isolate learning to local geometric cues alone, and observe that this learning can be blocked by, and block, learning about wall color information in a manner that is consistent with the model of spatial navigation proposed by Miller & Shettleworth (2007, 2008, 2013).

As noted previously, Pearce et al. (2004) trained rats to find a submerged platform in one corner of a rectangle-shaped arena, before placing rats into a kite-shaped arena. Following this shape transformation, rats preferentially navigated to the right-angled corner of the kite-shaped arena that shared the same local geometric cues that signaled the goal location in the rectangle-shaped, over the other right-angled corner. Interestingly, Pearce et al. (2004) did not observe a significant difference between first-choice visits to the correct right-angled corner and the acute corner in the kite-shaped arena. It is possible that rats’ preference for the acute corner reflects an unconditioned preference for small, dark corners of an environment. Cheng and Gallistel (2005), however, provided an alternative explanation. They argued that organisms extract the principal axis of the shape in which they are navigating, and search for a goal that is to one side of one end of this axis. As Cheng and Gallistel (2005) demonstrated, an organism could learn to navigate to the geometrically identical corners of a rectangle-shaped environment by relying on the principal axis of the rectangle. Importantly, when transferred to a kite-shaped arena, navigating to the previously rewarded side of the principal axis would leads rats to either the correct right-angled corner, or the acute corner. While this theory correctly predicts the behavior that was observed by Pearce et al. (2004), it has more difficulty explaining the first-choice data from our Experiment 1. Unlike the rats in the Pearce et al. (2004) experiment, our participants displayed a significant preference for searching in the correct right-angled corner only—a result that is more consistent with the notion that organisms navigate using local geometric cues (see also McGregor et al., 2006) than the principle axis.

In addition to navigation that is based upon the principal axes of environments, it is also important to consider the results of our experiments with reference to view-based theories of navigation. Historically, these theories have been applied to insect navigation (see Collett & Collett, 2002); however, view-based parameters have been used to model rodent navigation (e.g., Sheynikhovich, Chavarriaga, Strosslin, Arleo, & Gerstner, 2009), and have been applied to humans also (e.g., Epstein, 2008). Briefly, view-based theories suggest that, during navigation, organisms attempt to recover the view that was perceived from a goal location. For instance, a function can be used to determine the difference between the current image and the target image, and gradient descent can be used to minimize the discrepancy between these two panoramic images (e.g., Stürzl et al., 2008). Such view-based theories have successfully modeled how learning in an environment of one shape can transfer to an environment of another shape (Cheung et al., 2008), as we observed in Experiment 1. In addition to matching edges, it is not unreasonable to expect that humans may also match color information in stored views. If it is then assumed that the elements within a panoramic image are able to enter into cue competition, it would be possible for view-based theories to explain the results presented here. As suggested by Cheng, Huttenlocher, and Newcombe (2013), future experiments are required to determine the extent to which view-based processes contribute to human navigation. Data from children, however, suggest that view-based strategies do not principally control navigation in humans. In an experiment conducted by Lee and Spelke (2011), 3- and 4-year old children were able to navigate to the appropriate corners of a rectangle-shaped arena that was defined by low-height walls, but not when the rectangle-shaped arena was defined by a sheet on the ground. Importantly, the sheet provided salient high-contrast edges relative to the low-height walls and, thus, provided greater differences between the views of correct and incorrect locations. View-based matching theories, then, would expect the opposite pattern of results to what Lee and Spelke observed.

The experiments presented here were all conducted in a virtual-environment, in which participants were required to search for an invisible column. It might be reasonable to question the ecological validity of the effects we observed on two accounts. First, it could be argued that searching for an invisible column does not relate to real-world behavior. Despite this concern, we note that we have successfully used the same instructions used here in other experiments (e.g., Buckley, Smith, & Haselgrove, 2014, 2015a). Moreover, searching for an invisible goal is akin to searching for a Wi-Fi or mobile (cell) phone signal, and experiments in which we have asked participants to search for a hidden Wi-Fi signal have demonstrated comparable effects to those observed here (see Experiment 3 of Buckley, Smith, & Haselgrove, 2015b). Nevertheless, we acknowledge that our experiment was a computer-generated simulation of navigational behavior that may be different to that performed in real-world environments. Second, organisms receive vestibular, proprioceptive, and somesthetic inputs during real-world experiments, but not in virtual-reality experiments (Lavenex & Lavenex, 2010). It might, therefore, be argued that our participants used visual, and not navigational, systems to complete our task. However, effects observed in humans navigating in a virtual-world have also been reported in real-world experiments that have been conducted with animal subjects. For instance, the design of Experiment 1 (see also Lew et al., 2014), was based on an effect first observed in rats navigating in a water-maze (e.g., Pearce et al., 2004), and generated comparable results. Similarly, the blocking results observed in Experiment 2 (see also Wilson & Alexander, 2008) have been reported in studies conducted with rats in water mazes (e.g., Pearce et al., 2006). In addition, it has been demonstrated that the hippocampus is involved in navigation based on the boundary shape of an environment in rats (e.g., Horne, Iordanova, & Pearce, 2010), and this has also been observed in human participants navigating on the basis of the boundaries of a virtual environment (e.g., Doeller, King, & Burgess, 2008). Similarly, para-hippocampal regions have been shown to be implicated in learning about landmarks in rats (e.g., Kosaki, Poulter, Austen, McGregor, 2015) and also in humans learning about landmarks in a virtual environment (e.g., Doeller et al., 2008).

In the non-spatial literature, it has been observed that a change in context between Stage 1 and Stage 2 training attenuates blocking. For instance, in flavor aversion learning, the ability of a flavor cue to block learning about a second flavor cue is reduced by a change in context (Bonardi, Honey, & Hall, 1990). In the present Experiments 2 and 3, blocking was observed despite a change in the spatial context between Stage 1 and Stage 2. It is conceivable, then, that the magnitude of the blocking effects in Experiments 2 and 3 were attenuated because of the change in context. This suggestion must be treated with caution, though. The current experiments were not designed to address the effect of changing contexts; thus, we did not prepare an appropriate control condition in which participants experienced no context change. Additionally, in the experiments conducted by Bonardi, Honey, and Hall (1990), the flavor cues were presented to rats in two different cages; thus, the conditioned stimuli were discrete from the context in which they were presented. In our experiments, however, the context was defined by the shape and color of the arenas, and these cues were also the conditioned stimuli. As the arenas we used in Stage 1 and Stage 2 were designed such that they shared the same wall colors (Experiment 2), or the same local geometric cues (Experiment 3), it is debatable as to whether the shift between Stage 1 and Stage 2 in our experiments represents a context change in the manner reported by Bonardi et al. (1990).

To conclude, spatial learning based upon the shape of the environment transferred to an environment that was a different global shape, but which shared local geometric information. Moreover, learning about local geometric information was blocked by, and could block, learning about non-geometric wall colors. These results are difficult to reconcile with an analysis of spatial navigation that emphasizes the role of a global representation of environmental shape that is impervious to cue-competition (e.g., Cheng, 1986; Cheng & Gallistel, 2005; Gallistel, 1990). In contrast, the current results suggest that learning about local geometric cues occurs in a manner consistent with an associative model of spatial navigation (Miller & Shettleworth, 2007, 2008, 2013).

## Figures and Tables

**Table 1 tbl1:** The Number of Participants (out of 16) in Groups Rectangle-Kite and Kite-Rectangle That Visited the Correct, Incorrect, Acute, or Obtuse Corner First During the Same, and Different, Color Transfer Tests of Experiment 1

		Zone			
Group	Transfer test	Correct	Incorrect	Acute	Obtuse
Rectangle-kite	Same color	12	1	1	2
	Different color	12	0	2	2
Kite-rectangle	Same color	14	2	.	.
	Different color	12	4	.	.
*Note*. Dots in the acute and obtuse cells represent that these corners were not present at test for group kite-rectangle.
